# Automated Mobile Phone–Based Mental Health Resource for Homeless Youth: Pilot Study Assessing Feasibility and Acceptability

**DOI:** 10.2196/15144

**Published:** 2019-10-11

**Authors:** Angela C Glover, Stephen M Schueller, Dominika A Winiarski, Dale L Smith, Niranjan S Karnik, Alyson K Zalta

**Affiliations:** 1 Department of Psychiatry & Behavioral Sciences Rush Medical College Rush University Chicago, IL United States; 2 Department of Psychological Science University of California Irvine, CA United States; 3 Center for Behavioral Intervention Technologies Department of Preventive Medicine Northwestern University Chicago, IL United States; 4 Department of Psychology Olivet Nazarene University Bourbonnais, IL United States

**Keywords:** mental health, young adult, homelessness, telemedicine, treatment, mHealth, mobile phone

## Abstract

**Background:**

Youth experiencing housing instability have higher rates of mental health problems than their housed peers. Few studies have evaluated technological resources for homeless youth to determine how to effectively engage and reach them.

**Objective:**

The primary aims of this pilot study were to establish the feasibility (as measured by phone retention rates) and acceptability (ie, participant ratings of resources) of delivering automated mental health resources via smartphone technology.

**Methods:**

Youth aged 16 to 25 years (N=100) were recruited through homeless shelter agencies in the Chicago metropolitan area. Eligible participants completed a baseline assessment and received a smartphone with a 3-month data plan. The phone was preloaded with several apps designed to promote mental health wellness and provide real-time resources. One app specifically designed for this study, Pocket Helper 2.0, sent participants daily surveys and tips via push notification. The tips focused on coping and motivation, and the surveys assessed mood. This app also included an automated self-help system with brief cognitive behavioral interventions (5-10 min) and access to several interactive mobile tools, including a crisis text line, a telephone hotline, a crowd-based emotional support tool, and an app providing up-to-date information on social service and mental health resources for homeless youth in Chicago. Participants completed assessments at 3 and 6 months.

**Results:**

Some individuals (23%, 23/100) experienced problems with the phones (eg, theft, loss, and technological issues) throughout the study. Participant retention at the midpoint was moderate, with 48% (48/100) of youth responding to the 3-month surveys. At 6 months, only 19% (19/100) of the total sample responded to the end point survey. Overall, 63% (30/48) to 68% (13/19) of respondents at both time points reported benefiting from the intervention; however, participant usage and satisfaction varied with the different features. At both time points, participants reported receiving the most benefit from the daily tips and daily surveys. Daily tips that were most preferred by participants involved motivational tips related to overcoming struggles and making progress in life. Aside from the tips and surveys, the most used features were the app providing up-to-date resources and the automated self-help system. Interactive features, including the telephone hotline and crowd-based emotional support tool, were the least used features and were rated as the least beneficial.

**Conclusions:**

Automated mental health interventions seem to be an acceptable way to engage homeless youth in mental health support. The participants preferred fully automated features and brief interventions over features requiring interaction with others or more engagement. Future research should explore ways to retain homeless youth in interventions and evaluate the clinical impact of automated technology-based interventions for improving mental health.

**Trial Registration:**

ClinicalTrials.gov NCT03776422; https://clinicaltrials.gov/ct2/show/NCT03776422

## Introduction

### Background

#### Youth Homelessness

Each night, thousands of young people across the United States experience housing instability. Most recent statistics from 2018 indicate that as many as 36,361 unaccompanied youth are counted as homeless on a given night [1]. In Chicago specifically, it was estimated that 80,384 people experienced homelessness in 2016, 11,067 of whom were unaccompanied youth aged 14 to 24 years [2]. Youth experiencing homelessness have very specific mental health needs that often go unaddressed because of barriers to accessing care. One barrier is that young people experiencing homelessness often have to focus on emergent and immediate needs—finding housing, securing their belongings, and seeking employment—so, out of necessity, mental health needs become lower priorities. Traditional services tend to require scheduling an appointment in advance and having reliable transportation to an office or clinic, both of which present challenges for young people experiencing homelessness. Even when young people experiencing homelessness receive services, these often do not adequately address the various stressors and challenges associated with homelessness. Therefore, it is important to explore novel ways of reaching this population and of providing resources consistent with the needs identified by the youth.

#### Technology as Means of Homelessness Engagement

One avenue to reach young people experiencing homelessness might be through technology-based resources given the high levels of engagement with technology in this age group and the value of mobile technology as a resource for homeless individuals [3]. According to a 2019 survey by the Pew Research Center, 96% of US adults between the ages of 18 and 29 years own a smartphone [4], and 48% of them report that they go online *almost constantly* [5]. Importantly, technology access and use among individuals experiencing homelessness is also high, with approximately 44% to 62% of homeless individuals reporting ownership of a mobile phone [6]. One study that sampled 249 homeless individuals in an emergency department found that 70.7% of the sample owned a mobile phone [7], and another study sampling 169 homeless youth in an urban city found that 62% of them owned a mobile phone, although only 40% reported that they had a working phone [8]. Moreover, smartphone dependency, that is, those who have access to smartphones but not broadband internet access, is highest among the lowest income groups in the United States [4]. Access to mobile technology among individuals experiencing homelessness is particularly important because these devices act as a portal by which they connect to critical resources. Mobile phones may be the only way they can search for employment and other resources and stay connected with family members and care providers.

#### Technology & Mental Health

Technology-based interventions are increasingly being used in the medical field to increase access to care, including technology-based treatments for mental health issues such as anxiety, depression, and substance use [9]. These interventions have been structured around teletherapy, text messaging, and mobile apps. Research has shown that technology-based interventions are a promising way to deliver mental health treatment to various populations, including college students [10] and individuals with schizophrenia and bipolar disorder [11]. Technology-based mental health treatment has also been effective in clinical and nonclinical populations in primary care, emergency departments, and outpatient settings as well as in community settings [12,13]. It has been proposed that technology holds the potential to overcome disparities present in traditional health service delivery [14]. However, very few mobile interventions have been attempted with homeless youth. Existing technology-based interventions in this population have largely focused on reducing HIV risk behaviors [15,16]. Studies have also used technology as a method for improving the accessibility of case management and maintaining communication between homeless youth and social workers [17,18]. Collectively, these studies have shown that mobile technology holds promise for engaging youth in care by offering convenience and a source of connection. Here we seek to extend this broader line of research to attempt to develop a mental health intervention for homeless youth using mobile technology.

In our previous pilot study, 35 shelter-based homeless youth (aged 18-24 years) were given the opportunity to schedule 3 coaching sessions of 30 min each over the phone with a doctoral-level therapist over the course of 1 month [19]. Subjects were allowed to reach out to the study coach via text messaging during the intervention period. In addition, a mobile app was created for the study that sent participants a daily survey to assess sleep and stress, and a daily tip, which focused on various coping skills or motivational messages. This app was specifically geared toward youth. Satisfaction with the intervention was high, and most participants completed the 3 counseling sessions (57%). Conflicts between the youth’s availability and the coach’s schedule contributed to as many as 20% of youth being unable to benefit from the counseling sessions. In addition, this study found that the self-reported benefit of the automated tips in the study app was higher than the self-reported benefit of the counseling sessions. It is possible that participants may have liked the automated features such as the tips because they were readily available when they needed them.

Although the preponderance of evidence suggests that human support is a critical element of the most engaging and effective technological interventions [20-23], most recent research shows high rates of engagement and clinical benefit such as reductions in depression, anxiety, and other symptoms of psychopathology and increases in well-being, even from fully automated technological interventions [24-27]. These new interventions make use of emerging technologies such as virtual conversational agents or *chatbots* [27]. Therefore, it might be that newer fully automated technological interventions may be as impactful in engagement as those that include human support, especially for different subpopulations. Fully automated technological interventions could be appealing to youth experiencing homelessness because they are continuously available—they do not require appointments or scheduling during normal working hours and because developing trust with human supporters might present additional barriers. These are especially important factors to consider when designing apps or interventions for homeless youth whose schedules and circumstances are highly variable.

### Objectives

On the basis of participant feedback from the pilot study, and in an effort to provide the intervention to a larger group while addressing clinician-identified barriers, which included the difficulty of meeting youths’ needs outside of working hours, this study sought to evaluate the feasibility and acceptability of a fully automated mobile phone–based intervention for homeless youth. In expanding the intervention to a larger group, we expanded recruitment to youth accessing emergency overnight shelters and drop-in centers who often have even fewer options for accessing reliable mental health care. Although previous research has shown that fully automated interventions typically have lower engagement than human-supported interventions [28], with early withdrawal from the interventions and poor retention, the goal of this study was to provide youth with real-time mental health resources. We also sought to test a variety of different technology-based tools (push notifications, stand-alone apps, crisis text line, telephone hotline, and social network support tool) to determine which intervention modalities the youth preferred. The primary aims of this study were to (1) evaluate the acceptability of the interventions, as measured by participant satisfaction ratings collected at 3 months and 6 months, and (2) evaluate the feasibility of the interventions, as measured by participant retention and phone loss rates.

## Methods

### Participants

Participants for this pilot study were recruited from December 2017 to January 2019 from 2 homeless shelter agencies located in Chicago, Illinois. Potential participants were referred to the study by their case manager, responded to flyers distributed in shelters, or were recruited from in-person information sessions carried out by study staff in shelters. Interested youth were screened at the shelter by a member of the study staff.

Eligibility criteria for this study intervention included the following: (1) age 16 to 25 years, (2) English speaking, (3) experiencing housing instability as defined by “lacking a fixed, regular, and adequate nighttime residence OR whose primary nighttime residence is a shelter, institution, or a public or private place not designed for, or ordinarily used as, a regular sleeping accommodation for human beings,” sharing the housing of other persons because of loss of housing [or] economic hardship, frequent moves, poor housing quality (eg, living in severely overcrowded housing), or imminently leaving the foster care system [29], and (4) willingness and ability to comply with requirements of the study protocol. Exclusion criteria included (1) unwillingness to adhere to study procedures and (2) previous enrollment in the pilot study. General informational sessions about the study were held for youth at local homeless shelters. A total of 103 youth were screened, and 101 were enrolled in the program. In addition, 1 youth was ineligible because of age and 1 was uninterested after reviewing the informed consent form; 1 youth withdrew from the study after enrolling but before completing the baseline questionnaires and receiving the phone. Thus, the final study sample included 100 participants.

All participants were sent a set of midpoint surveys at 3 months. Of these 100 participants, 48 (48%) completed the surveys. Those who completed the 3-month surveys with valid data had their paid phone service and study participation extended for 3 additional months and were sent the same set of surveys at 6 months (end point). Of the 48 participants who had received the 6-month surveys, 19 (40%) completed the end point surveys.

### Procedures

This field trial was approved by the Rush University Medical Center institutional review board (IRB). If eligible, participants went through the informed consent process with a member of the study staff and then filled out a series of baseline assessments on an iPad. Under the Illinois Emancipation of Mature Minors Act (750 ILCS 30), a 16-year-old minor is mature enough to manage his or her own affairs. Thus, the Rush IRB granted permission for youth aged 16 to 17 years to consent for themselves without a parent or guardian. Baseline assessments collected information about demographics and trauma history. Although it is beyond the scope of this paper, it should be noted that data were also collected on a range of mental health symptoms. After completing these surveys, youth were provided with an Android smartphone (which was theirs to keep after the study was complete) with an activated 3-month, 5 GB per month data plan, a phone case, and headphones. Even if the participant already had a smartphone, they were given a new device and asked to use this device for the duration of the study. Participants were shown how to use a selection of the 15 apps downloaded on the phone and were given a handout describing the uses of all 15 apps (see Multimedia Appendix 1). Participants were then given tips on how to conserve cellular data and how to use the phone responsibly and safely in an urban space.

**Figure 1:**
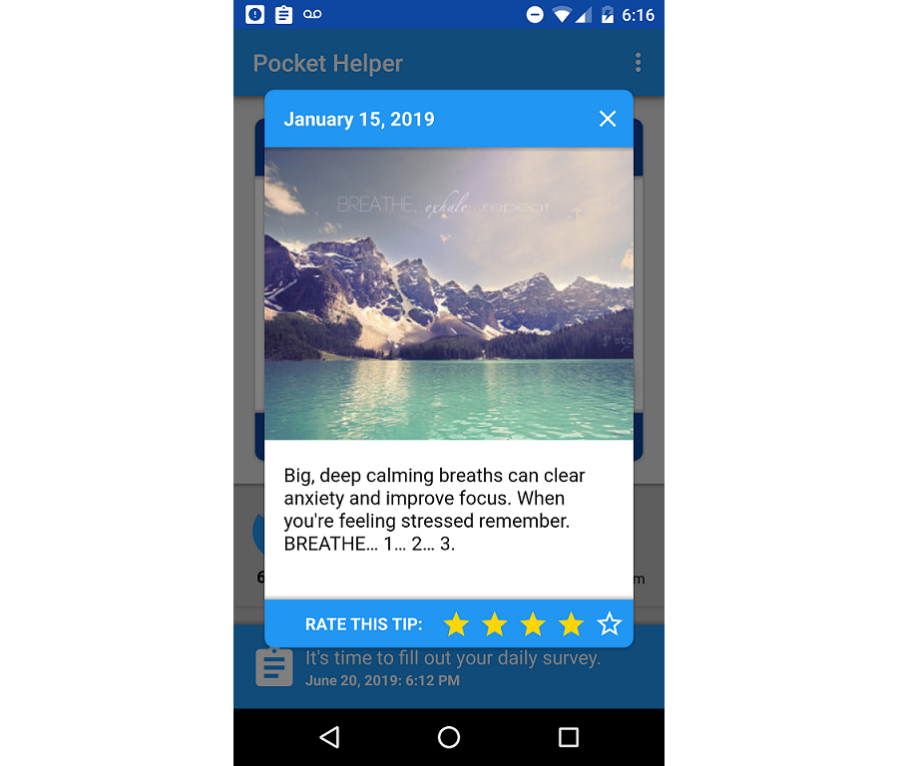
Example of a daily tip in the Pocket Helper 2.0 app.

Participants were asked to engage in 2 activities daily while participating in this study. First, participants were sent a daily survey via the Pocket Helper 2.0 app that asked them to rate their stress level for the previous day on a scale from 1 to 7, pick 3 emotions from a list of positive and negative emotions that most accurately described how they felt that day, and then briefly state the biggest challenge they faced in the past day. Participant engagement in the study was gauged by their completion of daily surveys. Good engagement was defined as completing at least 50% of daily surveys in every 2-week period of the study or 7 out of every 14 surveys. Good engagement was incentivized, and participants could earn a US $5 virtual Target gift card for every 2-week period that they had good engagement. This portion of the intervention was designed based on the principles of contingency management that has been shown to be effective in behavior change for youth [30]. Second, participants received a push notification for a daily tip sent to them via the Pocket Helper 2.0 app. The tips focused on mental health and provided various coping techniques and motivational messages (see Figure 1). Participants were asked to rate how much they liked each tip on a scale from 1 to 5 stars, with 5 being the highest rating of likability. The number of tip ratings was tracked but not incentivized. Participants were also not incentivized for using the other apps preloaded onto their phones.

Furthermore, 4 weeks before the 3-month midpoint date, participants were sent a link directing them to the midpoint survey. Participants were also sent a reminder to complete the survey via text or email each week up until the midpoint date. Surveys were sent out in advance because of the difficulty of engaging with these youth upon the first attempt to increase the likelihood that the surveys would be completed by the midpoint date. These surveys included a feedback questionnaire designed to assess the acceptability of different intervention components. If the participants completed the survey with valid responses (determined based on accurate responses to at least 4 of 6 validity items embedded in the survey), their paid phone and data service was extended for another 3 months. If they did not complete the surveys or did not get at least 4 validity items correct, participation in the program ended.

If participants received the 3-month extension, the program continued as described above. At 5 months, a set of end point surveys, identical to the midpoint surveys, were sent out. If participants completed the surveys and answered at least 4 validity items correctly, they received a virtual US $25 Target gift card. At the end of the 6-month study period, the phone company providing service, Sparrow Mobile, reached out to the participants with information on how they could continue their service with this provider. A member of the study team also reached out to the participants to offer other government resources for maintaining a phone service.

### Mobile Phone Apps

The following mobile apps were included on each phone given to participants.

#### Pocket Helper 2.0

Pocket Helper 2.0 is a mobile app designed specifically for this study. It is an updated version of an app that was developed for our previous pilot study [19]. The app offered several features, including a daily survey and a daily coping skills–focused tip (see Figure 1), sent to users via a push notification. Pocket Helper 2.0 also provided access to various platforms for participants to receive live emotional support (Koko, Illinois Warm Line, and Crisis Text Line, described in detail below) as well as an integrated support system that guides participants through brief cognitive behavioral interventions.

#### Koko

Koko is a mobile intervention designed to provide emotional support. It is a further iteration of the Panoply platform that was tested as a Web-based intervention to facilitate cognitive restructuring through crowdsourcing [31]. Koko provides access to a peer network that provides emotional support, including support leveraging cognitive behavioral principles [32]. Users do not interact with other users directly but through a chatbot. Koko screens all the messages for indicators that a user might be an imminent danger to himself or herself or others and automatically initiates a crisis protocol in these situations.

#### Illinois Warm Line

The Warm Line was developed by the Illinois Mental Health Collaborative for Access and Choice. Available Monday through Friday from 8 am to 5 pm, this is a telephone hotline that provides mental health support, mentoring, and advocacy from Peer and Family Support Specialists. There is no crisis support. This service is available for free throughout Illinois [33].

#### Crisis Text Line

Unlike the Warm Line, the Crisis Text Line provides 24x7 text-based support specifically for individuals in a crisis. The service is available for free throughout the United States. Individuals can receive support through text from a trained crisis counselor. Crisis counselors help individuals using empathetic listening and collaborative problem solving to come up with a safety plan for the texter [34].

#### Pocket Helper 2.0 Support System

The Pocket Helper 2.0 Support System is an automated system that provides brief (5-10 min) cognitive behavioral interventions, including strategies to promote relaxation, gratitude, emotion regulation, and effective goal setting.

#### IntelliCare Apps

IntelliCare is a modular treatment suite consisting of 13 *miniapps,* each focused on a singular behavior change technique drawn from cognitive behavioral therapy and positive psychology [35]. Examples of apps include Purple Chill, which provides exercises youth can do to build relaxation and meditation skills; iCope, which sends users self-authored inspirational messages in times of stress; and Thought Challenger, which teaches users to identify and restructure unhelpful negative thought patterns. See Multimedia Appendix 1 for a description of all IntelliCare apps.

#### StreetLight Chicago

StreetLight Chicago is a mobile app developed by Young Invincibles in collaboration with the Chicago Coalition for the Homeless with funding by the VNA Foundation. The app features up-to-date information on shelters, health clinics, emergency resources, and mental health services within the Chicago area [36].

### Assessment and Measurement

#### Demographics

This 41-item questionnaire, developed by the study team, assesses demographic characteristics (eg, age, sex, sexual orientation, race, ethnicity, educational status, employment status, and pregnancy or parenting status), homelessness status, health and mental health history, treatment history, current medical insurance, and access to mobile technology. This questionnaire was administered at baseline, midpoint, and end point.

#### Childhood Trauma

Possible trauma endured in childhood was assessed using 3 subscales (15 items) of the 28-item Childhood Trauma Questionnaire [37]. The selected scales were used to reduce participant burden and were focused on domains of interest. The 3 subscales contain 5 items each and assess physical, emotional, and sexual abuse. This self-reported measure asks participants to rate how often they experienced physical, emotional, and sexual abuse in childhood using a scale of 1 (never true) to 5 (very often true). Subscale scores range from 5 to 25, and total scores range from 15 to 75, with higher scores indicating a greater experience of childhood abuse. Clinically significant levels of abuse can be judged as follows on each subscale: physical (greater than 7), emotional (greater than 8), and sexual (greater than 5). This questionnaire was administered only at baseline to determine lifetime trauma exposure.

#### Anxiety

The computer-adaptive Patient-Reported Outcomes Measurement Information System (PROMIS) Bank V10 Anxiety measure [38] assesses symptoms of anxiety in the past 7 days. This is a reliable measure that has been validated in numerous populations [39]. Participants select 1 of 5 responses ranging from never to always. A *t* score of 50 reflects the average rating for the US general population, and every 10 points represent 1 SD from the mean.

#### Depression

The computer-adaptive PROMIS Bank V10 Depression measure [38] assesses symptoms of depression in the past 7 days. This is a reliable measure that has been validated in numerous populations [39]. Participants select 1 of 5 responses ranging from never to always. The scoring range for this measure is the same as the PROMIS Anxiety scale.

#### Feedback

The perceived benefit of the study was assessed using a 16-item questionnaire developed by the study team. This questionnaire asked participants to rate the overall study and specific intervention tools. Questions asked participants to respond on a 5-point Likert scale with responses ranging from 0 (not at all) to 4 (a lot), with an option for 5 (not applicable, did not use feature). Qualitative responses on which features were liked / disliked and why youth preferred certain features were also collected. This questionnaire was administered only at midpoint and end point.

## Results

### Statistical Analysis

Data for this study were collected in REDCap, a secure Web app for managing online surveys. Descriptive analyses were run in SPSS 22 Premium to determine frequencies, means, and standard deviations of baseline demographic data and feedback data at the 3-month midpoint and 6-month end point of the study for participants who completed the assessments with valid data.

### Data Exclusion

Baseline data for 1 participant were lost because of internet connectivity issues at the shelter where they were enrolled. Data were analyzed for the remaining 99 participants.

### Sample

A total of 100 youth consented and were enrolled in the field trial. The average age of the sample was 20.03 years (SD 1.83, range 16-24). On average, the participants had been homeless 3.4 times (SD 3.5) over their lifetime and 2.3 times (SD 2.7) in the past year. The average age of the participants at the first episode of homelessness was 17.0 years (SD 3.9). Furthermore, the mean length of the current episode of homelessness was 8.2 months (SD 13.3), and on average, the longest episode of homelessness was 13.7 months (SD 17.9). At the time of enrollment, 35 participants (35%) were enrolled in school and 27 (27%) were employed. In addition, 6 (6%) participants were currently pregnant and 18 (18%) were a parent of a dependent-aged child. Notably, 41 (41%) of the participants already owned a mobile phone at the time of enrollment. Furthermore, 70 (71%) of the youth reported having received therapy or counseling for mental health issues in their lifetime and 38 (38%) reported being currently engaged in therapy or counseling. As illustrated in Table 1, most participants in our sample also reported enduring various forms of abuse in childhood. At baseline, average self-reported anxiety levels (mean 60.1, SD 9.7) and depression levels (mean 58.6, SD 10.2) were both elevated compared with the general population. See Table 2 for additional demographic characteristics.

**Table 1:** Self-reported abuse history of an urban sample of unstably housed youth based on Childhood Trauma Questionnaire score. (Childhood Trauma Questionnaire scores indicating a clinically significant level of abuse are detailed in the assessment description above.)

Type of abuse	Mean (SD)
Physical abuse	12.2 (6.4)
Emotional abuse	15.1 (6.9)
Sexual abuse	8.9 (6.3)
Total score	36.2 (16.7)

**Table 2:** Demographic characteristics of an urban sample of unstably housed youth (n=99).

Characteristic		Value, n (%)
**Gender**		
	Male	53 (54)
	Female	39 (39)
	Male to female transgender	3 (3)
	Female to male transgender	4 (4)
**Sexual orientation**		
	Straight or heterosexual	75 (76)
	Gay or lesbian	9 (9)
	Bisexual	8 (8)
	Other	5 (5)
	Refused	1 (1)
	Don’t know	1 (1)
Ethnicity (Hispanic or Latino)		23 (23)
**Race**		
	Black or African American	57 (58)
	American Indian or Alaskan Native	2 (2)
	Native Hawaiian or Pacific Islander	2 (2)
	White non-Hispanic	10 (10)
	Mixed	19 (19)
	Other	5 (5)
	Refused	2 (2)
	Don’t know	2 (2)

### Feasibility

Overall, 48 of the 100 participants (48%) completed the midpoint assessments, and all participants who completed these assessments provided valid data. Of the 48 who had received end point assessments, 19 (39%) completed the measures and all provided valid data. Although this retention rate is not high, it is consistent with rates reported from previous studies evaluating automated mental health interventions [21].

We evaluated whether there were differences in baseline characteristics of those who completed the assessments compared with those who did not complete the assessments. Those who did not complete the midpoint survey reported significantly lower levels of childhood emotional abuse at baseline (mean 13.47, SD 6.77) than those who completed the midpoint survey (mean 16.85, SD 6.67; *d*=0.50, *P*=.01). Those who did not complete the midpoint survey were also less likely to own a mobile phone at baseline (χ^2^_1_=6.3; *P*=.01) and have medical insurance at baseline (χ^2^_1_=5.9; *P*=.02) compared with those who completed the midpoint survey. At end point, those who did not complete the survey were more likely to have been hospitalized for a psychological problem in their lifetime than those who completed the survey (χ^2^_1_=5.30; *P*=.02). Notably, these differences were no longer significant after Bonferroni correction for multiple testing. There was no association between completion status of the 2 assessments and other demographic characteristics (sex, sexual orientation, age, race, ethnicity, children, time spent homeless, school enrollment, employment status, receipt of psychotherapy, and psychotropic medication usage) or anxiety and depression symptom severity at baseline. Given the large number of tests conducted, these findings suggest that those who responded to the surveys were largely similar to those who dropped out of the study; however, there may be some meaningful differences between the groups, and the survey results should be interpreted in light of this potential bias.

**Figure 2:**
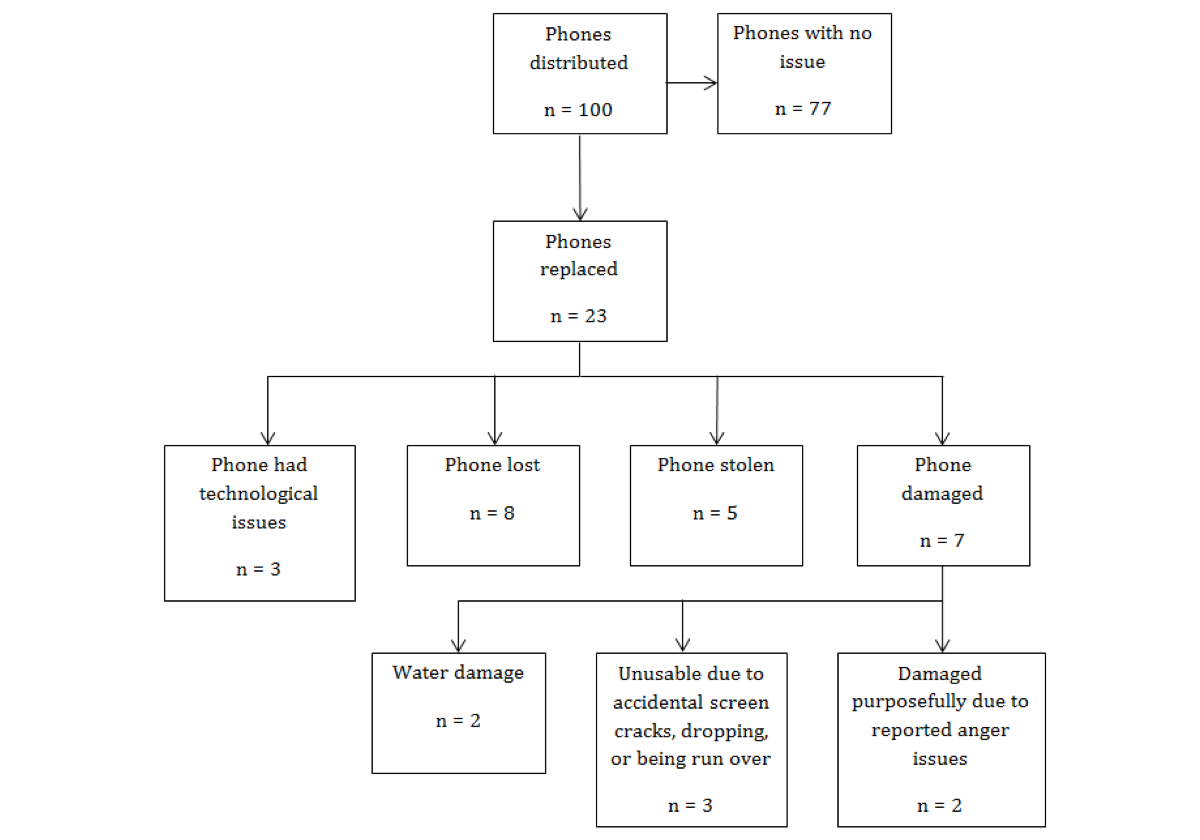
Phone distribution and reasons for replacement.

Over the course of the study, 23 study phones were replaced (23%) across the 100 participants. Phones were replaced for various reasons including loss, theft, damage, and technical issues (see Figure 2).

### Acceptability

#### 3 Months (Midpoint)

At 3 months, overall satisfaction with the study was high. Of the 48 participants who completed the midpoint assessments, 35 (73%) would recommend the study to somebody else. In addition, 30 participants (63%) reported a moderate-to-high amount of benefit from the study. If participants indicated that they benefited in any amount from the study, they were asked to describe how. There were a number of reasons they stated including learning new coping skills and receiving motivation from the daily tips. The most common themes were being able to reflect on and contextualize their emotions via the daily survey and having access to a working cellphone.

The features included in the intervention were used to varying degrees at the 3-month midpoint. The StreetLight app was the most used feature, with 79% (38/48) of participants reporting that they had used the feature. The Pocket Helper 2.0 Support System was used almost as much, with 77% (37/48) of participants reporting usage. There was moderate usage of the IntelliCare apps (29/48, 60%) and the Crisis Text Line (28/48, 58%). Koko and the Illinois Warm line were the least used features. Just under half of the participants reported using Koko (23/48, 48%) and fewer reported using the Illinois Warm Line (20/48, 42%).

**Figure 3:**
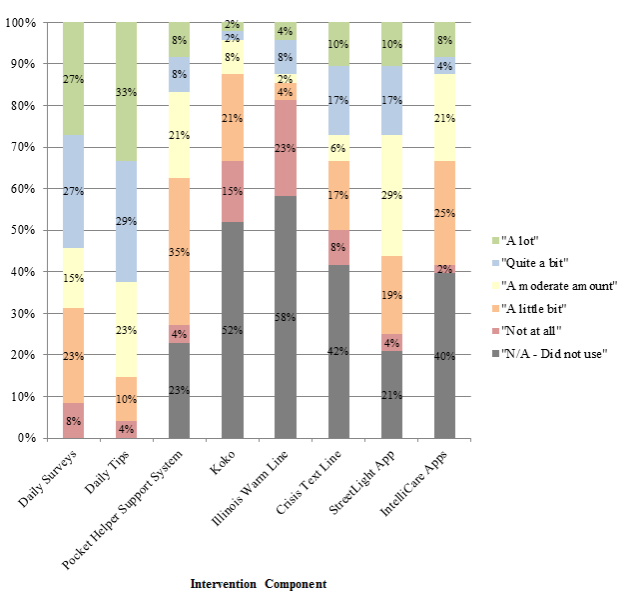
Self-reported benefit of intervention features at the 3-month midpoint. N/A: not applicable.

Figure 3 illustrates the perceived benefit ratings for each study component featured within Pocket Helper 2.0 and the separate StreetLight Chicago and IntelliCare apps. These data take into account all 48 participants who completed the midpoint survey, including those who indicated that they did not use a particular feature. The daily tips and daily surveys were reported to be the most helpful features with 85% (41/48) and 69% (33/48) of participants reporting that they benefitted at least a moderate amount, respectively. The StreetLight app was at least moderately helpful for 56.3% of participants (27/48), whereas the Pocket Helper 2.0 Support System, IntelliCare Apps, and Crisis Text Line were found to be at least moderately helpful for 33% to 38% of participants. Koko and the Illinois Warm Line were reported to be the least beneficial features, with only 13% to 15% of individuals reporting at least moderate benefit for both features.

Participants were also asked to select the feature they liked the most and the feature they liked the least in the Pocket Helper 2.0 app. Table 3 displays the percentage of people who selected each feature as being the most or least liked feature at the midpoint. The daily tips were reported to be the most liked feature (22/48, 46%), with the daily surveys close behind (21/48, 44%). The Illinois Warm Line was the least liked feature (13/48, 27%), followed by the daily surveys and Koko (9/48, 19%). Notably, there was much greater consensus on the most liked features compared with the least liked features. It is also interesting to note that the daily surveys dichotomized, with some youth reporting that they liked this feature and other saying that they disliked this feature.

**Table 3:** Self-reported most and least liked features in Pocket Helper 2.0 app. (3 months, n=48; 6 months, n=19.)

Pocket Helper 2.0 feature	Most liked feature at 3 months, n (%)	Least liked feature at 3 months, n (%)	Most liked feature at 6 months, n (%)	Least liked feature at 6 months, n (%)
Daily surveys	21 (44)	9 (19)	7 (37)	3 (16)
Daily tips	22 (46)	5 (10)	8 (42)	2 (11)
Pocket Helper 2.0 Support System	3 (6)	8 (17)	2 (11)	3 (16)
Koko	1 (2)	9 (19)	N/A	6 (32)
Illinois Warm Line	1 (2)	13 (27)	1 (5)	4 (21)
Crisis Text Line	N/A	4 (8)	1 (5)	1 (5)

#### 6 Months (End Point)

In total, 19 of the youth who qualified to continue post-3-month assessment had completed the 6-month end point assessment at the time of study analysis. Satisfaction at 6 months was still high, with 16 participants (84%) reporting that they would recommend the study to somebody else. A majority of participants (13/19, 68%) reported that they benefited at least *a moderate amount*. Similar to the midpoint of the study, participants who benefited from the study were asked to describe how. There were multiple reasons stated, including being able to use the daily tips in times of stress and benefiting from tracking daily actions in one of the IntelliCare apps. Again, the most common themes were motivation to reflect on day-to-day emotions via the daily survey and ownership of an active phone.

**Figure 4:**
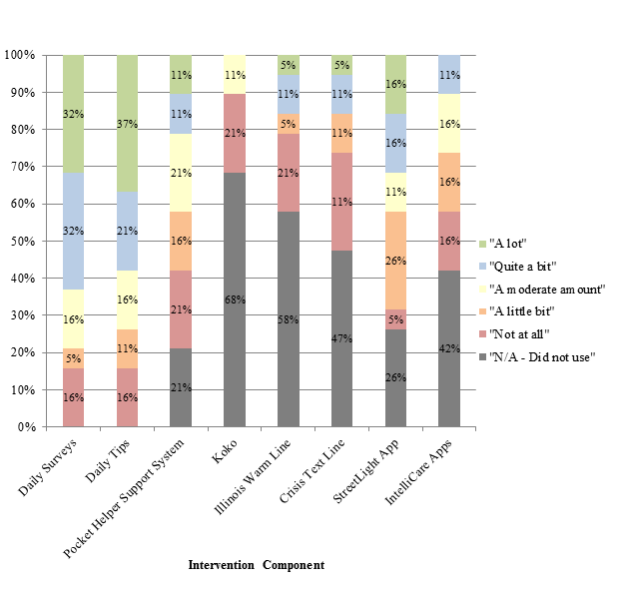
Self-reported benefit of intervention features at the 6-month end point. N/A: not applicable.

At the 6-month end point of the study, participants were asked again to report on their usage of the various apps. The Pocket Helper 2.0 Support System continued to be the most used feature, with 79% of participants (15/19) indicating usage. Also consistent with the 3-month data, StreetLight Chicago was the second most used feature with a similar usage rate of 74% (14/19). The usage of the IntelliCare apps and the Crisis Text Line remained similar to each other, although the usage rates of these dropped slightly compared with the 3-month time point. Eleven participants (57%) reported using the IntelliCare apps and 10 (53%) used the Crisis Text Line. The Illinois Warm Line and Koko were still the least beneficial features. However, the usage of the Illinois Warm Line remained the same while usage of Koko dropped noticeably from the 3-month time point. Eight participants (42%) reported using the Illinois Warm line, while only 6 participants (32%) reported using Koko. Only two of the 19 participants (11%) reported that they did not use any features other than the daily tips and surveys. Figure 4 illustrates the perceived benefit of each tool at the 6-month assessment. Similar to the midpoint data, the 6-month data included participants who reported that they did not use the various features. Consistent with the 3-month data, daily tips and daily surveys were rated as the most helpful features with 14 (74%) and 15 (78%) participants reporting at least a moderate benefit from the features, respectively. The StreetLight app, Pocket Helper 2.0 Support system, and IntelliCare apps were found to be at least moderately helpful for 26-42% of participants. Notably, the perceived benefit of the Crisis Text Line decreased from the midpoint to the end point with 16% (3/19) reporting at least a moderate benefit. The same number of participants reported a benefit from the Illinois Warm Line at 6 months. Similar to the midpoint, Koko was found to be the least helpful feature, with only 11% (2/19) of participants reporting at least a moderate benefit from the feature.

Table 3 also displays the percentage of participants who selected each feature as being the most or least liked feature at the end point. Participants overwhelmingly indicated the daily surveys (7/19, 37%) and daily tips (8/19, 42%) as their favorite features of the study app, Pocket Helper 2.0. In addition, 2 participants (11%) reported that their favorite feature was the in-app Pocket Helper 2.0 Support System, whereas 1 participant (5%) reported that they liked the Crisis Text Line and the Illinois Warm Line the most. Participants rated Koko (6/17, 32%) and the Illinois Warm Line (4/17, 21%) the lowest. Furthermore, 3 participants (16%) liked the daily surveys or Pocket Helper 2.0 Support System the least, 2 (11%) liked the daily tips least, and 1 (5%) liked the Crisis Text Line least.

#### Daily Tip Ratings

As the daily tips were rated as the most favorable and beneficial intervention feature, the specific tip ratings were analyzed to identify whether any patterns emerged. A pool of 49 tips was created to be included in Pocket Helper 2.0 (See Multimedia Appendix 2 for a list of all tips). Youth were sent a different tip at random every day for the first 49 days until they had received every tip. Starting at day 50, tips were delivered based on the youth’s rating of the tip, such that tips rated to be most liked over the first 49 days were sent more frequently starting on day 50. Therefore, participants’ responses to the initial presentation of each tip in the first 49 days were analyzed.

On average, participants who had at least 1 tip rating rated 14.61 tips during the first 49 days (SD 12.39, minimum=1, maximum 48, median=10). Table 4 portrays the average ratings for the 6 highest-rated and the 6 lowest-rated tips. Tips were rated on a scale of 1 to 5, with 5 being the highest rating of likability. As illustrated in Table 4, the lowest-rated tip had a mean rating of 4.00 out of 5, suggesting that, overall, all tips were rated highly by the youth. Notably, the highest rated tips acknowledged challenges and provided motivational messages about overcoming and moving past struggles.

**Table 4:** Tip rating descriptive statistics (in order of preference).

Tip		n^a^	Mean (SD)	95% CI
**Highest-rated tips**				
	Don’t put off until tomorrow what you can get done today.	34	4.71 (0.62)	4.51-4.92
	“It’s not whether you get knocked down, it’s whether you get up.” [Vince Lombardi]	24	4.71 (0.55)	4.49-4.93
	We all have setbacks. It’s okay to be disappointed, but don’t let them break you.	29	4.62 (0.68)	4.37-4.87
	No one can predict the future. Sometimes we have to wait and see what happens. Try not to spend too much time in the future. Stay in the present moment.	28	4.57 (0.92)	4.23-4.91
	Progress requires patience. Few things that are very important to us can be achieved in one day, but if you stick to the plan you’ll get there.	27	4.57 (0.88)	4.25-4.90
	Motivation can be contagious. Surround yourself with people who are working hard towards their goals, and hold each other accountable.	23	4.57 (0.79)	4.24-4.89
**Lowest-rated tips**				
	Checking something off your to-do list every day can help you feel accomplished, even if it’s small. Pick one task to achieve for the day.	29	4.17 (0.91)	3.84-4.49
	Tell someone you appreciate them. Showing gratitude to the people who are important to us can make YOU feel great!	24	4.17 (1.05)	3.75-4.59
	Just because you think something doesn’t make it true. If it’s not helping you, see if you can find another way of looking at it, or let it go.	30	4.15 (1.23)	3.73-4.57
	It’s always hard to establish a new skill. Remember that it takes practice when you try something new. Try it out for a week and then decide if it helps.	24	4.13 (1.33)	3.59-4.66
	How’s your day going today? Check in with yourself and see how you’re feeling. What are you feeling in your body? How’s your mood? Whether you’re feeling happy, sad, or anywhere in between, I’m sending you a pick-me-up!	32	4.00 (1.27)	3.56-4.44
	Don’t struggle with what you can’t change, but don’t think you have no control at all over your environment. Focus on what IS in your control.	29	4.00 (1.33)	3.51-4.49

^a^n indicates the number of participants that rated the tip upon its first presentation (within the first 49 days).

## Discussion

### Principal Findings

Overall, our findings suggest that an automated mobile phone–based intervention can be a promising way of engaging homeless youth around mental health. We were able to successfully recruit 100 homeless youth into the study, including many youth who were accessing emergency overnight shelters and drop-in centers (34% of current sample, 34/100) that traditionally serve more transient youth with less access to mental health services than their shelter-based peers. Overall, 77% of the study sample appeared to keep and maintain their cellphones in good condition over the 6-month study period. Throughout the entire study, only 20 of the 100 distributed cell phones were reported to be lost, stolen, or damaged, and an additional 3 phones had technical issues. Collectively, these data support the feasibility of a fully automated intervention over an extended period in a population of homeless youth.

Overall, 63% of participants at 3 months and 68% of participants at 6 months reported that they received at least moderate benefit from the intervention. Thus, the intervention appeared to be well-liked by those who maintained participation. Though participants who stayed engaged through the midpoint or end point reported that they benefited from the study, the actual retention rates from baseline to end point were lower than predicted. Overall, 48% of the total sample (48/100) completed the 3-month assessment, and only 19% of the total sample (19/100) completed the 6-month assessment. As previously noted, the youth in this study experienced greater housing instability than those from the interim housing programs recruited into the pilot study discussed above [19]. This pilot study also lasted 1 month compared with 6 months; both of these reasons might explain the much higher rates of retention (33/35) observed in the pilot study compared with the fully automated intervention described in this study. It is also possible that the participants in this study had more competing day-to-day priorities that could have contributed to the attrition rate. Further research is needed to explore what factors may affect engagement with mobile interventions for homeless youth.

Despite the high rate of mental health problems reported in this population, it is important to note that only 38 participants (38%) indicated that they were currently engaged with therapy services at baseline, and many of the youth in this study had access to these services at their shelter locations. By contrast, over 75% of participants who responded to the surveys reported that they used our brief self-help system at the 3-month follow-up. This means that although we were not able to retain as many people as we would have liked, our rate of engagement at the study midpoint was greater than the rate engaged in traditional mental health services at the time participants enrolled in the study. This again suggests that an automated, mobile-based tool kit might be a viable option for engaging a greater number of homeless youths in mental health care. It is important to note that the Pocket Helper 2.0 app was specifically designed for homeless youth based on initial input from these youth [40] and was refined based on feedback received during a previous pilot trial [19]. Involving the target end users in the design process from the start is a standard practice in the co-design process. Co-design may be especially important for underserved or marginalized populations such as homeless youth to ensure that tools are truly developed to meet their needs [41]. Co-design has been used successfully in other populations to develop tools tailored to their needs and challenges [42]. Therefore, it is possible that engagement was relatively high because the app was tailored to the needs of these youth and that not all mobile interventions would be equally acceptable to homeless youth.

One goal of this study was to evaluate how youth used the various feature modalities of the intervention. The features reported to be of the greatest benefit were the daily tips and daily surveys, which all youth received as a push notification to their phones. In fact, at both 3 and 6 months, participants overwhelmingly rated these 2 features of the Pocket Helper 2.0 app most favorably, with 69% to 85% reporting at least moderate benefit from these features across both follow-up time points. Previous studies have shown that the very act of self-reflection and self-monitoring can be therapeutic in a treatment context [12,43,44]. It is important to note that the surveys were incentivized with small payments, which may have affected the acceptability ratings for this intervention feature. However, the tips were also rated very favorably without any incentive, suggesting that participants may be responding positively to the interactive style of engagement with these features. The tip ratings also seem to suggest that participants particularly liked receiving tips that were motivational and encouraged them to overcome struggles and work toward progress. Given that homeless youth are often isolated with limited social support [45], having this type of motivational feature may be important for engaging the youth. Future directions of this project should more carefully evaluate specific reasons why participants enjoyed these features of the Pocket Helper 2.0 app so much more than other apps provided to them in this study.

The StreetLight app, Pocket Helper 2.0 Support System, IntelliCare apps, and Crisis Text Line were used by the majority of participants; however, it is clear that the perceived benefit from these features did not match the perceived benefit from the daily tips and surveys. These features all involved minimal-to-no direct human interaction but still required participants to be proactive in engaging with them, unlike the tips and surveys that were sent as a daily push notification. Out of the 4 aforementioned features, the StreetLight app and Pocket Helper 2.0 Support System were the most used features (74%-79% usage over the study), though not all those who used these features found them to be beneficial (37%-56% reported at least moderate benefit). Notably, both the StreetLight app and Pocket Helper 2.0 Support System were specifically designed for homeless individuals, whereas the IntelliCare apps and Crisis Text Line were designed without a specific subpopulation in mind. It is also noteworthy that the brief cognitive behavioral self-help tool was as engaging and appealing to participants as an app that provided real-time information about local resources such as food and shelter. This suggests that homeless youth in this study placed equal value on both basic needs and mental wellness, and further suggests that by removing logistical barriers to care, youth are able to and want to prioritize mental health.

With respect to the IntelliCare apps and Crisis Text Line, a minority of participants (16%-33%) found these features to be at least moderately beneficial. Both the Crisis Text Line and the IntelliCare apps require repeat engagement for maximum benefit, whereas the StreetLight app and Pocket Helper 2.0 Support System are able to provide support in a single interaction. Moreover, the daily tips and surveys were pushed to participants’ phones automatically and did not require the youth to initiate engagement with the app. Overall, these findings suggest that participants preferred tools that required minimal investment.

Koko and the Illinois Warm Line were consistently among the least used features, with 48% and 42% of responders indicating usage of these features at 3 months, respectively. The usage of Koko dropped to 32% at 6 months, whereas the usage of the Illinois Warm Line stayed consistent at 42%. The reported benefits of the Illinois Warm Line and Koko were also low compared with other features. Less than 15% of youth reported at least a moderate benefit for each feature at the midpoint. By the end point, only 3 of 19 (15%) participants reported at least a moderate benefit from the Illinois Warm Line and 2 of 19 (11%) participants reported at least a moderate benefit from Koko. Youth may prefer the features such as the Crisis Text Line over the Warm Line because it allows them to engage via text messaging rather than over the phone, consistent with previous studies that have shown that texting is the preferred method of communication in this age group [46,47]. It is also possible that the youth disliked the features that required more social interaction (ie, Illinois Warm Line and Koko) because of poor experiences with the mental health system in the past and a general sense of mistrust of telling *strangers* about their problems [48,49]. Trust difficulties coupled with these adverse treatment experiences in the past may increase youth’s skepticism toward apps requiring social interaction with others.

It is important to note that the usage and likability of the features varied between participants and between time points. For example, at 3 months, the daily survey was rated as the second most liked feature and was also tied as the second least liked feature. This may indicate some degree of dichotomization of participant preferences and should give pause in developing a *one-size-fits-all* approach. Although an option is to provide all possible tools with the hopes that it might provide something desirable to the maximum number of individuals, it is unclear whether there are any adverse impacts associated with providing youth with tools they do not like or do not find beneficial. One of the advantages of technology-based interventions is the option for customization or digital precision approaches. Future studies should also seek to evaluate adaptive iterations of this intervention, such that these apps can be tailored to the specific mental health needs of the youth using them or that specific features may be more relevant at certain times of a youth’s life. Future research should also explore the extent to which the inclusion of less favorable features may or may not detract from the usage of more favorable ones.

The results of our study suggest that participants were actively engaged in our intervention. At the same time, approximately one-third of the participants at the midpoint and end point reported that simply having access to a working phone was one of the most important benefits of study participation. Not surprisingly, addressing digital poverty in this population not only increases access to mental health care services but also allows for greater independence in other areas of youth’s lives (eg, being able to contact a potential employer), which could also positively impact mental health. This possibility should be explored in greater detail in future follow-up studies with homeless youth.

Although a thorough discussion of the ethics of providing interventions using a technology platform is well beyond the scope of this paper, it is important to highlight the need to weigh the pros and cons of utilizing this intervention in lieu of formal therapy. The authors do not assert that this intervention should replace traditional care, but rather see it as a bridge to future treatment. When the alternative is no mental health care, providing youth with the tools they need to address their mental health concerns in *real time* is an ethical and clinically sound strategy.

Finally, the low retention rates in this study warrant further exploration. Although some recent research studies have demonstrated that automated interventions without a human component can yield high levels of engagement and do not necessarily increase the risk of attrition [24,26], many studies have found that human support leads to higher rates of engagement [50]. Thus, although our participants reported liking these automated features, this does not mean the features were sufficient to keep them engaged. Of course, there is also the possibility of *happy abandonment* insofar as youth might have felt they got everything they could expect from these features and saw no incremental benefit of continuing to use them. It is important to note that previous work has not been done in homeless youth, and it is not known which variables unique to this population may maximize the likelihood of engagement with a fully automated intervention. It is possible that sustained engagement with an intervention that does not yield immediately measurable results is difficult, particularly when there are so many competing priorities. Future research should explore ways to increase engagement of homeless youth in a mobile phone–based mental health intervention over a longer time or evaluate whether a brief, more targeted intervention may be more efficacious and sustainable in this population.

### Limitations

One major limitation of this study is selection bias. The youth in this study were all connected to mental health and/or case management support through the shelter networks in the Chicago area. Future iterations of this work should also try to reach youth living on the streets without access to interim housing or drop-in shelters. Related to this point is that feedback about the intervention was only obtained from youth who demonstrated some level of continued engagement, and it is unknown why youth lost at follow-up assessment time points discontinued study participation. A better understanding of the variables that contribute to retention among homeless youth would allow us to more successfully tailor future iterations of technology-based interventions for this population.

As the data collected in this study were primarily quantitative, less is known about the specific reasons why youth preferred certain tools (eg, why the daily tips and surveys emerged as the most highly rated components of the intervention). In particular, because the surveys were incentivized, we were unable to determine whether the payment affected the youths’ acceptability ratings and whether this feature would have been rated less favorably without the incentive. Conducting focus groups or individual interviews with homeless youth in the future would allow for a more careful assessment of app preferences, a better understanding of the perceived benefits of study participation, and participant-driven suggestions for future iterations of fully automated interventions in this population.

### Conclusions and Future Directions

The fully automated, mobile phone–based mental health intervention evaluated in this study demonstrates both feasibility and acceptability in providing mental wellness tools to underserved homeless youth with limited access to traditional care. Overall, it appears that participants tended to prefer both automated and self-help features, as compared with ones involving more direct human interaction. This may result from this work being done in homeless youth as youth, especially digital natives who have been raised in the digital age, may be more comfortable connecting to people and receiving information and support through digital means. Most youth have used the internet to find health information or download a health app, and many of them use it to connect to other people regarding health concerns [51]. The fact that tips and surveys were clearly favored suggests that these youth prefer both brief and passive interactions with technology. In fact, youth reported continued usage of the Pocket Helper 2.0 Support System over the course of the study, and even preferred it over the StreetLight app, which provides citywide resources related to basic needs for homeless youth. Collectively, these results suggest that youth prefer digital tools that engage with them and that require only brief interactions for benefit. A critical next step is to evaluate the perceived clinical benefits of this intervention. As previously mentioned, mental health data were collected at each time point of the study, and future research should evaluate whether participation in this fully automated intervention yields reduction in self-reported mental health difficulties and improvements in overall mental wellness.

## Appendix

Multimedia Appendix 1 Guide to apps provided to participants in study.

Multimedia Appendix 2 Tip rating descriptive statistics (in order of preference; greatest to least).

## Abbreviations

IRB: institutional review board
PROMIS: Patient-Reported Outcomes Measurement Information System
