# Supplementary material for: Automated Mobile Phone–Based Mental Health Resource for Homeless Youth: Pilot Study Assessing Feasibility and Acceptability
Source: JMIR Ment Health. 2019 Oct 11;6(10):e15144. doi: 10.2196/15144 (PMC6915810; doi:10.2196/15144)
Supplement: Multimedia Appendix 1 [file mental_v6i10e15144_app1.pdf]

The smartphone you receive will also contain multiple apps that can help you develop skills to manage stress and make positive changes in your life. Here is an idea of what some of these apps can be useful for:

Pocket Helper 2.0: Use this app to complete your daily surveys and rate your daily tips. Also use this app to access other tools, including:

> **Pocket Helper 2.0 Support System:** Use this tool to find help getting support in hard times, or to get tips and ideas for improving wellness, coping, and goal setting

Koko: Receive support from this social network when you're dealing with a stressful situation

> **Illinois Warm Line:** Receive emotional support over the phone

> **Crisis Text Line:** If you feel overwhelmed by your stress or negative emotions, text with someone here until you feel that you are safe

StreetLight Chicago: Find up to date information on shelters, health clinics, emergency resources, and mental health resources in the Chicago area

IntelliCare Apps: Use these 13 apps to learn how you can take control of your behaviors. Gain tools and develop skills to improve your ability to sleep, relax, manage negative thoughts, utilize social support, etc.

Aspire – Identify your values and get guidance on actions you can take that reflect those values

Boost Me – Get in the moment suggestions of activities you can do to boost your mood when stressed, sad, bored, or feeling badly about yourself

Daily Feats – Helps you to stay engaged and productive every day, and to track and celebrate your accomplishments

Day to Day – Focuses on teaching different mood-boosting or problem-solving skills each week for five weeks

iCope – Learn how to anticipate and cope with stressful situations, and create aspirational and reassuring messages for yourself

MoveMe – Helps you get and stay active with simple how-to videos and lessons on how to increase motivation

My Mantra – Helps you to develop and use simple phrases and images that can help you to notice and remember positive things in your daily life

Purple Chill – Teaches you mindfulness relaxation skills

Slumber Time – Helps you understand your sleep patterns and routines so that you can make changes to improve your sleep

Social Force – Helps you improve your mood by encouraging you to increase positive contact with supportive people in your life

Thought Challenger – Helps you improve your mood, decrease anxiety, and feel better by teaching you how to recognize and change unhelpful thought patterns

Worry Knot – Teaches you how to decrease your emotional response to worry and helps you distract yourself from your tangled thinking

IntelliCare Hub – Manage all of your apps in one place
