# Supplementary material for: Automated Mobile Phone–Based Mental Health Resource for Homeless Youth: Pilot Study Assessing Feasibility and Acceptability
Source: JMIR Ment Health. 2019 Oct 11;6(10):e15144. doi: 10.2196/15144 (PMC6915810; doi:10.2196/15144)
Supplement: Multimedia Appendix 2 [file mental_v6i10e15144_app2.pdf]

Tip rating descriptive statistics (in order of preference; greatest to least)

Tip	M	SD	95% CI
Don't put off until tomorrow what you can get done today.	4.71	0.62	4.51-4.92
It's not whether you get knocked down, it's whether you get up." – Vince Lombardi	4.71	0.55	4.49-4.93
We all have setbacks. It's okay to be disappointed, but don't let them break you.	4.62	0.68	4.37-4.87
No one can predict the future. Sometimes we have to wait and see what happens. Try not to spend too much time in the future. Stay in the present moment.	4.57	0.92	4.23-4.91
Progress requires patience. Few things that are very important to us can be achieved in one day, but if you stick to the plan you'll get there.	4.57	0.88	4.25-4.90
Motivation can be contagious. Surround yourself with people who are working hard towards their goals, and hold each other accountable.	4.57	0.79	4.24-4.89
Take time out to enjoy the day. I hope this one makes you laugh!	4.54	0.89	4.25-4.84
Keep track of your progress every day. It will help you see how far you've come.	4.52	0.64	4.28-4.76
Anger can be a sign that something needs to change. Make sure to make a smart change though. Don't act quickly, slow down, take your time, and make a plan.	4.50	0.98	4.16-4.84
There is rarely only one way to fix a problem. Try many solutions to see what works.	4.50	0.91	4.15-4.85
Keeping an optimistic view requires you to be kind to yourself. What's one nice thing you can do for yourself today?	4.50	0.92	4.16-4.84
The only thing that sets successful people apart from anyone else is their refusal to give up. Find a way to pull yourself back up when you feel discouraged.	4.48	0.83	4.18-4.78
Anxiety isn't always bad. Anxiety is our body's signal that something is going on. Learn to listen to your body. What is your anxiety telling you?	4.48	1.08	4.07-4.89
Reaching your goals is hard work. What is really important to you? Pick goals that move you toward the things that are the most important to you. This will make the hard work worth it.	4.46	0.74	4.19-4.74
Everyone struggles with self-confidence sometimes. When you find yourself comparing yourself to others, remember that everyone is on their own path. What is one nice thing you can say to yourself today?	4.46	0.88	4.14-4.79
90% of success is just showing up. What will you show up to today?	4.46	0.72	4.17-4.75

What is the most important thing you need to do this week? What are the steps you need to take to get that done? Write down these small goals and see if you can begin checking them off.	4.45	0.89	4.14-4.76
People don't know how to help you, unless you ask for what you need. Learning to ask for what you need is important in building relationships and reaching your goals. Try asking someone for something you need today.	4.44	0.8	4.14-4.75
You miss 100% of the shots you don't take.	4.41	1.18	3.98-4.84
Try to appear confident even when you feel unsure of yourself. Fake it until you make it!	4.41	1.27	3.95-4.88
It's okay to be sad, but don't let sadness overcome you. Think of doing something that usually makes you feel better, and give it a try. Like talking to a friend or going for a walk. Try to push yourself, even if you don't feel up to it in the moment.	4.40	1.1	4.01-4.79
It's easy to isolate yourself from others when you're feeling down. We all do it. Try reaching out to someone you trust today, and ask their advice.	4.40	0.91	4.04-4.76
Everyone has insecurities. Focus on what you like about yourself.	4.40	0.76	4.10-4.70
Behind every failure is a lesson. Take it as an opportunity to learn something new.	4.40	0.89	4.08-4.72
Making plans to meet your goals can be helpful, but don't get too wrapped up in them. Sometimes the best way to make a change is to start doing instead of planning.	4.39	1.03	4.01-4.77
When practicing guided imagery, remember to incorporate all 5 senses (sight, sound, smell, taste, touch) to make the picture as vivid as possible. Take any distracting noises (e.g., the sound of an air conditioner) and build them into your image to make it even more powerful.	4.37	0.88	4.04-4.70
Always have something that brings you happiness on hand. It could be a picture, some music, your favorite gum... Use it when you're feeling low.	4.37	0.93	4.02-4.72
Make sure your goal is within your control. You are able to choose how you behave, respond, and interact with others. You are NOT able to control the actions of others. Pick a goal based on something YOU can change.	4.36	1.03	4.01-4.71
Progressive muscle relaxation is a skill that has been shown to improve mood and decrease stress. Try taking a few moments out of your day to try this skill.	4.35	0.93	3.97-4.73
What are you grateful for today? Even if today is tough,	4.35	1.09	3.93-4.77

think about one thing that you appreciated today. Maybe it was the sunshine, a friend, or a song that you like. People who shift their outlook toward gratitude are happier, healthier, and more successful.			
When you're feeling discouraged, try to remember not just WHAT you're trying to achieve, but WHY.	4.33	1.1	3.98-4.69
Sometimes it is all about perspective. What will you choose to focus on today?	4.32	0.87	4.02-4.63
Everyone feels overwhelmed sometimes. Try to get a little bit done each day, but make sure you save time to recharge, too!	4.31	1.12	3.88-4.74
Not sure where to start? Pick a long-term goal and try working backwards to figure out what you can do now.	4.29	0.98	3.92-4.65
To reach a goal, start small. Tackling too much all at once is hard and can make you feel overwhelmed. Figure out where to start and do that before you take the next step.	4.27	1.17	3.85-4.69
When worries and negative thoughts crowd your mind, practice your skills. Try relaxation, guided imagery exercises, or soothing music.	4.27	1.05	3.89-4.64
Think of a time you felt accomplished. Write it down, and when you're feeling unmotivated remind yourself that you can do what you set your mind to.	4.24	0.93	3.88-4.60
We all feel alone sometimes. Text someone who cares about you, and remember that we're all in this together.	4.23	1.11	3.76-4.69
Think about all the things that make a "good day" for you. Try to do at least one of those today. How did you feel before, and how did you feel after?	4.21	1.25	3.71-4.71
Remember, that even when you're having a difficult moment, day, or week, that it will not last forever. Take a deep breath and think of something you've overcome in the past. Remind yourself that you can get through this.	4.21	1.29	3.74-4.68
It's easy to get distracted. Make a list of things that are important to you. When anything else starts to upset you or get in the way, remember this list.	4.21	1.05	3.83-4.59
Big, deep calming breaths can clear anxiety and improve focus. When you're feeling stressed, remember. BREATHE... 1... 2... 3.	4.19	1.09	3.81-4.57
A good night's sleep helps people feel happier. Try to get 7-9 hours by getting into bed on time and avoiding exercise, video games, caffeine, and nicotine before bed.	4.18	1.16	3.79-4.58
Checking something off your to-do list every day can help you feel accomplished, even if it's small. Pick one task to achieve for the day.	4.17	0.91	3.84-4.49

Tell someone you appreciate them. Showing gratitude to the people who are important to us can make YOU feel great!	4.17	1.05	3.75-4.59
Just because you think something doesn't make it true. If it's not helping you, see if you can find another way of looking at it, or let it go.	4.15	1.23	3.73-4.57
It's always hard to establish a new skill. Remember that it takes practice when you try something new. Try it out for a week and then decide if it helps.	4.13	1.33	3.59-4.66
How's your day going today? Check in with yourself and see how you're feeling. What are you feeling in your body? How's your mood? Whether you're feeling happy, sad, or anywhere in between, I'm sending you a pick-me-up!	4.00	1.27	3.56-4.44
Don't struggle with what you can't change, but don't think you have no control at all over your environment. Focus on what IS in your control.	4.00	1.33	3.51-4.49
